# Association between *Interleukin-1B* C-31T Polymorphism and Obesity in Japanese

**DOI:** 10.2188/jea.JE20081015

**Published:** 2009-05-05

**Authors:** Koji Suzuki, Takashi Inoue, Atsumi Yanagisawa, Asami Kimura, Yoshinori Ito, Nobuyuki Hamajima

**Affiliations:** 1 Department of Public Health, School of Health Sciences, Fujita Health University, Toyoake, Aichi, Japan; 2 Department of Preventive Medicine/Biostatistics and Medical Decision Making, Nagoya University Graduate School of Medicine, Nagoya, Japan

**Keywords:** interleukin-1beta, obesity, polymorphism, Japanese

## Abstract

**Background:**

Recent studies have revealed a close relationship between obesity and polymorphism in the inflammation gene. However, the association between interleukin-1beta (IL-1β) and obesity remains controversial. We therefore investigated the association between *IL-1B* C-31T polymorphism and obesity in Japanese.

**Methods:**

The participants were 802 inhabitants (281 men and 521 women) of Japan, aged 39 to 88 years, who attended a health examination in 2003. Body height, weight, waist and hip circumferences, and body fat percentage were measured. The *IL-1B* C-31T polymorphism was genotyped by polymerase chain reaction with confronting 2-pair primers. The association between *IL-1B* C-31T genotypes and various indices of obesity was then investigated. The confounding factor-adjusted odds ratios (OR) and 95% confidence intervals (CI) for obesity were calculated for each *IL-1B* C-31T genotype by using unconditional logistic regression analysis.

**Results:**

Among male carriers of the CT and TT genotypes, the ORs for high body fat percentage were 2.58 (95% CI, 1.17–6.34) and 2.81 (1.17–7.33), respectively, as compared to carriers of the CC genotype (*P* for trend = 0.037). Among women, carriers of the TT genotype had significantly higher ORs for high BMI (OR, 2.13; 95% CI, 1.25–3.67) and large waist circumference (2.49; 1.37–4.66), as compared to women with the CC genotype (*P* for trend = 0.005 and 0.004, respectively).

**Conclusions:**

The *IL-1B* C-31T polymorphism is associated with obesity in Japanese. Men and women with the TT genotype of *IL-1B* C-31T had a higher risk for obesity than those with the CC genotype.

## INTRODUCTION

Interleukin-1beta (IL-1β) is an important mediator of inflammation and is produced by a variety of cells, including macrophages and epithelial cells.^1^ It has been reported that genotype differences in *IL-1B* affect IL-1β protein secretion.^2^^,^^3^ The T allele of *IL-1B* C-31T forms a TATA box, which is suspected of enhancing gene expression.^4^ As compared to other genotypes, carriers of the -31CC genotype had significantly lower IL-1β mRNA levels in gastric mucosa, and an *IL-1B* promoter assay showed that the -31T promoter was associated with a 10-fold increase in activity, as compared to the -31C promoter.^3^

IL-1β is thought to have a role in the control of energy homeostasis, the suppression of adipose differentiation, and the expression and activity of lipoprotein lipase,^5^ and therefore may be involved in the regulation of body fat. It has been reported that mice with IL-1 receptor antagonist deficiency experience a decrease in fat mass.^1^ Moreover, abnormality of IL-1 and IL-6 in mice causes severe early-onset obesity.^6^ Several studies have investigated the association between *IL-1B* C-31T polymorphism and obesity in humans.^7^^,^^8^ Strandberg et al^7^ reported that *IL-1B* C-31T polymorphism did not correlate with fat measurements. However, their more recent study showed that *IL-1B* C-31T polymorphism was associated with body fat.^8^ Therefore, the association of *IL-1B* C-31T polymorphism with obesity remains controversial.

The association between *IL-1B* C-31T polymorphism and obesity has not been investigated in Japanese. We therefore investigated the associations between *IL-1B* C-31T genotypes and indices of obesity among a population of adult Japanese.

## PARTICIPANTS AND METHODS

### Study participants

Health examinations of inhabitants aged 39 years or older have been performed in Hokkaido, Japan since 1982.^9^ The study population comprised 864 participants (309 men, 555 women) who attended such a health examination in August 2003. We excluded 60 participants who did not agree to participate and 2 participants whose polymorphism was not determined. The remaining 802 participants (281 men, 521 women; age range, 39 to 88 years) were eligible for the present analysis. This study was approved by the Nagoya University Graduate School of Medicine Ethics Committee (Approval number 48).

### Methods

In the health examination, body height, weight, waist and hip circumferences, and body fat percentage were measured when the participant was in a fasting state, and wearing light clothes and no shoes. Body mass index (BMI) was calculated as body weight (kg) divided by height (m) squared and waist-to-hip ratio (WHR) was calculated as waist circumference (cm) divided by hip circumference (cm). Body fat percentage was measured using a commercially available body fat scale (Tanita TBF-305, Japan) that utilizes foot-to-foot bioelectrical impedance analysis. Analysis of the impedance values from this device revealed an intraday coefficient of variation (CV) of 0% to 6% and a between-day CV of 2% to 4%.^10^ Fasting serum lipid levels were measured using an autoanalyzer (JCA-RX20, Nihon Denshi Co., Ltd., Tokyo, Japan) on the day of the health examination.

Trained nurses administered a questionnaire regarding health and daily lifestyle habits, which included smoking status (current smoker, former smoker, or never smoker), alcohol consumption (current drinker, former drinker, or never drinker), frequency of physical activity (little, 1 to 2 hours/week, 3 to 4 hours/week, or 5 hours/week or more), and history of major illness.

### Genotyping

DNA was extracted from residual whole blood using a BioRobot EZ1 (QIAGEN Group, Tokyo, Japan). *IL-1B* C-31T polymorphism was genotyped by PCR-CTPP (polymerase chain reaction with confronting 2-pair primers).^11^

### Statistical analysis

All statistical analyses were conducted using statistical software (JMP ver. 7.0, SAS Institute, USA). The Hardy–Weinberg equilibrium, which indicates an absence of discrepancy between genotype and allele frequencies, was checked using the chi-square test. The associations between genotype and continuous variables, including age and serum levels of lipids, were tested by analysis of variance (ANOVA) and the Tukey–Kramer test. Age-adjusted anthropometric measurements and indices of obesity were examined using analysis of covariance. We defined obesity as a BMI of 25 or higher, which is in agreement with the guidelines of the Japan Society for the Study of Obesity.^12^ Epidemiologic studies have shown that Japanese participants with a BMI of 25 or higher had an increased risk for all-cause mortality and coronary heart disease.^13^^,^^14^ In the present study, high weight, waist circumference, WHR, and body fat were defined as the highest quartile by sex (weight: 69.5 kg in men and 60.2 kg in women; waist circumference: 89.0 cm in men and 80.0 cm in women; WHR: 0.934 in men and 0.843 in women; percent body fat: 25.9% in men and 34.9% in women). Odds ratios (OR) adjusted for age, smoking status, alcohol consumption, and frequency of physical activity with 95% confidence intervals (CI) were estimated by unconditional logistic regression analysis. A probability value less than 0.05 was considered statistically significant.

## RESULTS

The frequencies of the *IL-1B* -31 C and T alleles in study participants were 0.47 and 0.53, respectively. The CC, CT, and TT genotypes of *IL-1B* C-31T were present in 173 (21.4%), 411 (51.4%), and 218 (27.2%) participants, respectively. This distribution was within the Hardy–Weinberg equilibrium (*P* = 0.41).

Characteristics of the study participants by sex and genotype are shown in Table 1. The distribution of genotypes did not significantly differ by sex. Among both sexes, there was no significant difference in age, smoking status, alcohol consumption, or physical activity for the 3 *IL-1B* C-31T genotypes. Among women, carriers of the TT genotype had a significantly higher level of serum total cholesterol than did carriers of the other genotypes.

**Table 1.** Characteristics of study participants by *IL-1B* C-31T genotype

		Male				Female			
			
		CC	CT	TT	*P* value	CC	CT	TT	*P* value
Number		61	145	75		112	266	143	
	(%)	(21.7)	(51.6)	(26.7)		(21.5)	(51.1)	(27.4)	
Age*	y	65.1 ± 9.4	62.9 ± 10.9	61.8 ± 10.7	0.176^‡^	59.9 ± 10.3	60.2 ± 9.5	60.8 ± 10.8	0.749^‡^
Smoking status					0.937^§^				0.594^§^
​ Never	*n*	14	33	17		89	218	114	
	(%)	(23.0)	(22.8)	(22.7)		(79.5)	(82.0)	(79.7)	
​ Former	*n*	30	66	38		9	28	15	
	(%)	(49.2)	(45.5)	(50.7)		(8.0)	(10.5)	(10.5)	
​ Current	*n*	17	46	20		14	20	14	
	(%)	(27.9)	(31.7)	(26.7)		(12.5)	(7.5)	(9.8)	
Alcohol consumption					0.484^§^				0.183^§^
​ Never	*n*	21	55	29		91	228	121	
	(%)	(34.4)	(37.9)	(38.7)		(81.3)	(85.7)	(84.6)	
​ Former	*n*	7	7	7		0	7	4	
	(%)	(11.5)	(4.8)	(9.3)		(0.0)	(2.6)	(2.8)	
​ Current	*n*	33	83	39		21	31	18	
	(%)	(54.1)	(57.2)	(52.0)		(18.7)	(11.7)	(12.6)	
Physical activity					0.284^§^				0.240^§^
​ ≥2 h/wk	*n*	45	117	54		94	209	121	
	(%)	(73.8)	(80.7)	(72.0)		(83.9)	(78.6)	(84.6)	
​ <2 h/wk	*n*	16	28	21		18	57	22	
	(%)	(26.2)	(19.3)	(28.0)		(16.1)	(21.4)	(15.4)	
Total cholesterol*	mg/dL	201.3 ± 29.8	208.1 ± 33.0	210.3 ± 25.6	0.207^‡^	210.3 ± 31.0	218.3 ± 35.6	222.0 ± 28.2	0.016^‡^
HDL-cholesterol*	mg/dL	55.0 ± 15.5	53.6 ± 11.7	53.1 ± 11.1	0.657^‡^	62.4 ± 14.2	61.5 ± 14.0	62.2 ± 12.7	0.808^‡^
Triglyceride^†^	mg/dL	95.1	99.7	97.8	0.828^‡^	81.0	81.7	89.1	0.123^‡^
		(68.0–124.5)	(70.0–131.5)	(67.0–132.0)		(62.5–103.7)	(59.0–106.0)	(67.0–114.0)	

*Data are expressed as means ± standard deviation.

^†^Data are expressed as geometric means and 25th–75th percentiles in parentheses.

^‡^One-way analysis of variance.

^§^Chi-square test.

HDL-cholesterol: high-density lipoprotein cholesterol.

Table 2 shows a comparison of age-adjusted anthropometric measurements and indices of obesity by *IL-1B* C-31T genotype. Male carriers of the TT genotype had a significantly higher body weight, higher BMI, and higher body fat percentage than carriers of the CC genotype. Waist circumference and WHR were significantly higher in female carriers of the TT genotype than in carriers of the CC genotype. Body weight in female carriers of the TT genotype was higher than in those with the CC genotype, but the difference was not significant.

**Table 2.** Age-adjusted* mean and standard error of anthropometric measurements and obesity indices, by *IL-1B* C-31T genotype

			Male			Female	
			
		CC	CT	TT	CC	CT	TT
Number		61	144	75	112	264	143
Height	cm	163.1 ± 0.7	162.9 ± 0.5	163.6 ± 0.6	151.0 ± 0.5	151.9 ± 0.3	152.0 ± 0.4
Weight	kg	62.2 ± 1.2	64.6 ± 0.8	66.0 ± 1.1^†^	53.7 ± 0.8	55.4 ± 0.5	56.2 ± 0.7
BMI	kg/m^2^	23.4 ± 0.4	24.3 ± 0.3	24.7 ± 0.4^†^	23.5 ± 0.3	24.0 ± 0.2	24.4 ± 0.3
Waist circumference	cm	81.3 ± 1.2	83.1 ± 0.8	84.7 ± 1.1	72.9 ± 0.8	74.7 ± 0.5	76.0 ± 0.7^‡^
Hip circumference	cm	92.4 ± 0.7	94.0 ± 0.5	94.6 ± 0.6	91.6 ± 0.6	92.7 ± 0.4	93.5 ± 0.5
Waist-to-hip ratio		0.879 ± 0.008	0.882 ± 0.005	0.894 ± 0.007	0.794 ± 0.005	0.805 ± 0.003	0.811 ± 0.005^†^
Body fat percentage	%	20.9 ± 0.7	23.1 ± 0.4^†^	23.5 ± 0.6^‡^	29.8 ± 0.6	30.9 ± 0.4	31.1 ± 0.5

*Adjusted for age by using analysis of covariance.

^†^*P* < 0.05, ^‡^*P* < 0.01 (Significant difference versus CC genotype, Tukey–Kramer test).

BMI: body mass index.

Table 3 shows the confounding factors-adjusted OR and 95% CI, by genotype, for participants with the highest values on indices of obesity. As compared to male carriers of the CC genotype, the ORs for high body fat percentage for male carriers of the CT and TT genotypes were 2.58 (95% CI, 1.17–6.34) and 2.81 (1.17–7.33), respectively (*P* for trend = 0.037). Among women, carriers of the TT genotype had significantly higher ORs for high BMI (OR, 2.13; 95% CI, 1.25–3.67) and high waist circumference (2.49; 1.37–4.66), as compared to carriers of the CC genotype (*P* for trend = 0.005 and 0.004, respectively).

**Table 3.** Odds ratios and 95% confidence intervals for obesity, by *IL-1B* C-31T genotype

	Male				Female			
		
	CC	CT	TT	*P* for trend	CC	CT	TT	*P* for trend
Weight, ≥75th percentile	1.00	1.73 (0.81–3.95)	1.81 (0.78–4.40)	0.207	1.00	1.20 (0.70–2.08)	1.46 (0.82–2.67)	0.197
BMI, ≥25	1.00	1.67 (0.87–3.30)	1.35 (0.64–2.88)	0.508	1.00	1.47 (0.90–2.42)	2.13 (1.25–3.67)	0.005
Waist circumference, ≥75th percentile	1.00	1.89 (0.91–4.21)	1.94 (0.85–4.64)	0.145	1.00	1.94 (1.11–3.49)	2.49 (1.37–4.66)	0.004
Waist-to-hip ratio, ≥75th percentile	1.00	1.00 (0.49–2.08)	1.32 (0.60–2.97)	0.473	1.00	1.36 (0.79–2.39)	1.47 (0.81–2.72)	0.230
Body fat percentage, ≥75th percentile	1.00	2.58 (1.17–6.34)	2.81 (1.17–7.33)	0.037	1.00	1.47 (0.86–2.59)	1.52 (0.84–2.79)	0.204

Odds ratios are adjusted for age, smoking status, alcohol consumption, and frequency of physical activity.

Data are shown as odds ratios with 95% confidence intervals in parentheses.

BMI: body mass index.

## DISCUSSION

The present study showed that *IL-1B* C-31T genotype was associated with obesity among a sample of the Japanese population. The men and women with the TT genotype of *IL-1B* C-31T had a higher risk for obesity than those with the CC genotype. After adjustment for age, smoking status, alcohol consumption, and physical activity, the odds ratios for high body fat percentage among men, and high BMI and waist circumference among women, remained significant. We hypothesize that these differences result from sex differences in fat distribution.

The T allele at -31 forms a TATA box, which is believed to enhance gene expression.^4^ A recent study reported that IL-1β mRNA expression was elevated in TT genotype carriers, as compared to CC and CT genotype carriers, for the *IL-1B* C-31T polymorphism.^15^ It was therefore suggested that TT genotype carriers had a higher production of IL-1β than did carriers of the CC or CT genotype. However, another study^16^ found no association between *IL-1B* C-31T polymorphism and serum levels of C-reactive protein (CRP). We believe that it is not easy to determine the effect of polymorphism on serum levels of CRP, because serum CRP levels are associated with a number of factors, such as age, smoking habits, alcohol consumption, stress, blood pressure, and obesity.^17^

IL-1β suppresses lipoprotein lipase (LPL) expression and activity.^5^ LPL is an enzyme that hydrolyzes triglyceride-rich lipoproteins and is expressed in a variety of tissues, including adipose tissue and muscle.^18^ It has been reported that overexpression of LPL reduced fat accumulation and insulin resistance in rabbits fed high-fat diets,^19^ indicating that elevation of LPL may protect against obesity. Thus, increased IL-1β production may be involved in the development of obesity.

IL-1β also decreases insulin-induced glucose transport in adipocytes^20^ and induces insulin resistance in adipocytes. Because insulin resistance also suppresses LPL activity and is an impediment to the catabolism of very low-density lipoprotein (VLDL), production of VLDL in the liver is increased and hyperlipidemia occurs. These long-term effects on lipid metabolism may play a role in the onset of obesity. Because IL-1β production is increased in carriers of the TT genotype of *IL-1B* C-31T, as compared to other genotypes, LPL expression and activity may be lower in carriers of the TT genotype. We suggest that this mechanism is one reason for the effect of *IL-1B* C-31T polymorphism on obesity. In this study, carriers of the TT genotype of *IL-1B* C-31T had higher serum cholesterol levels than those with the CC genotype, particularly so in female carriers. There were higher levels of triglycerides and lower levels of HDL-cholesterol among carriers of the TT genotype in both sexes, but the differences were not significant, most likely because serum triglyceride and cholesterol levels are affected by other factors, such as diet, smoking and drinking habits, and physical activity.

Studies have shown that IL-β has an antiobesity effect and that IL-1 is involved in the leptin-induced suppression of feeding.^21^ In addition, studies of knockout mice have indicated that immune systems with IL-1 have an antiobesity effect.^1^^,^^6^ These findings, however, do not correspond to those of the present study. Because we did not determine the caloric intake of study participants, we could not ascertain whether the *IL-1B* C-31T polymorphism was associated with the amount of food eaten. Although there have been no reports of an association between *IL-1B* polymorphism and the amount of food consumed, further study will be necessary to examine this association.

There have also been cross-sectional studies that investigated the association between *IL-1B* C-31T polymorphism and obesity.^7^^,^^8^ Strandberg et al^7^ noted that *IL-1B* C-31T polymorphism did not correlate with body fat percentage in 1068 young white men aged 18 to 20 years. However, they did note that, among 3014 Swedish men aged 69 to 81 years, carriers of the CC genotype had higher total fat mass, as measured with dual-energy X-ray absorptiometry.^8^ Although they could not explain this discrepancy, they suggested that it was related to age difference, in keeping with the age-dependent effects of IL-1 deficiency in rodents.^6^

The reasons for the discrepancy between previously published reports and our results are unknown, but IL-1β production is influenced by the other *IL-1B* polymorphisms and by factors such as genetic background and epigenetic conditions. Three diallelic polymorphisms in *IL-1B* have been reported, all representing C-T base transitions, at positions -511, -31, and +3954 (formerly +3953) base pairs from the transcriptional start site.^4^ Because *IL-1B* -31C and -511T are almost in complete linkage disequilibrium and display a similar pattern of phenotype associations, one of these polymorphisms might serve as a marker for the other. The T allele of *IL-1B* C3953T was associated with a 4-fold increase in the production of IL-1β, as compared with the C allele.^22^ However, the minor allele (T) of *IL-1B* C3954T is rare in Japanese, as compared with whites.^23^ In addition, the frequency of the *IL-1B* 3953T allele in Japanese is much lower (CC, 92.2%; CT, 7.8%; TT, 0.0%)^24^ than in whites in Poland (CC, 56.4%; CT, 36.8%; TT, 6.8%)^4^ and Finland (CC, 51.5%; CT, 41.0%; TT, 7.5%).^23^

This is the first report of an association between the *IL-1B* C-31T polymorphism and obesity among the Japanese population. However, the mechanism underlying this association is unclear. Because participants with the T allele of *IL-1B* C-31T were more likely to be obese than those with the C allele, persons with the T allele may need to take special care to adhere to a healthy lifestyle.
